# Identification of a Rye Spring Mutant Derived from a Winter Rye Variety by High-Altitude Environment Screening Using RNA Sequencing Technology

**DOI:** 10.3390/genes15050572

**Published:** 2024-04-29

**Authors:** Yanying Wang, Yixuan Liu, Chengqun Yu, Shizhan Chen, Yankun Li, Lina Wei, Junxi Wu, Jianping Yang

**Affiliations:** 1 Key Laboratory of Biodiversity and Environment on the Qinghai–Tibetan Plateau, School of Ecology and Environment, Ministry of Education, Tibet University, Lhasa 850000, China; yan.ying.wang@outlook.com (Y.W.); yixuan.liu@foxmail.com (Y.L.); 2 Lhasa Plateau Ecosystem Research Station, Key Laboratory of Ecosystem Network Observation and Modeling, Institute of Geographic Sciences and Natural Resources Research, Chinese Academy of Sciences, Beijing 100101, China; yucq@igsnrr.ac.cn; 3 Center for Crop Genome Engineering, State Key Laboratory of Wheat and Maize Crop Science, College of Agronomy, Henan Agricultural University, Zhengzhou 450046, China; szchen@henau.edu.cn (S.C.); ykli@stu.henau.edu.cn (Y.L.); lnwei@stu.henau.edu.cn (L.W.)

**Keywords:** *Secale cereal* L., Tibet, winter–spring characteristic, RNA-seq

## Abstract

*Wintergrazer-70* and *Ganyin No1* are high-yield forage varieties suitable for cultivation in high-altitude areas of Tibet (4300 m above sea level). *Ganyin No1* was developed from *Wintergrazer-70*, with the latter serving as its parent variety. *Ganyin No1* was identified as a spring variety, and subsequent RNA sequencing was conducted. RNA sequencing analysis identified 4 differentially expressed genes related to vernalization and 28 genes related to photoperiod regulation. The Sc7296g5-i1G3 gene is related to the flowering inhibition of rye, which may be related to the phenotypic difference in the *Ganyin No1* variety in winter and spring. This finding provides valuable insights for future research on *Ganyin No1*, especially in addressing feed shortages in Tibet during winter and spring.

## 1. Introduction

The vernalization process is pivotal in wheat, serving as a crucial determinant for distinguishing between winter and spring varieties [1]. The common methods for winter–spring identification in China primarily involve spring sowing in the field. In the Huanghuai region, the heading rate during the second stage of spring sowing is a fundamental indicator for determining winter and spring wheat varieties [2,3]. The determination of wheat’s spring and winter varieties is regulated by the activation and inhibition mechanisms of vernalization genes *Vrn1* and *Vrn2* [4]. The control of vernalization is regulated by one or several dominant alleles at the *Vrn-1* loci [3], with homologous genes located on the long arms of chromosomes 5A, 5B, and 5D, respectively [5,6]. The photoperiodic genes, namely *Ppd-D1* (*Ppd1*), *Ppd-B1* (*Ppd2*), and *Ppd-A1* (*Ppd3*), are located on chromosomes 2D, 2B, and 2A, respectively [7]. The presence of both the photoperiodic gene *Ppd-D1a* and the vernalization gene *Vrn-D1* alleles in winter wheat, under winter conditions and specific genotypes, substantially improves the consistency of flowering timing [7].

Varieties containing the *Vrn-B1* gene are typically classified as spring or weak spring varieties, while those with recessive alleles for *Vrn-A1*, *Vrn-B1*, and *Vrn-D1* are predominantly winter or semi-winter varieties [8]. The dominant *Vrn-D1* allele is associated with weak winter or weak spring characteristics [9]. The combination of recessive alleles *Vrn-A1*, *Vrn-B1*, *Vrn-D1*, and *Vrn-B3* results in winter or semi-winter phenotypes [10]. Combining vernalization gene markers with traditional winter–spring identification methods may enhance the accuracy of variety classification. The photoperiodic recessive gene *Ppd-D1b* is typically found only in winter varieties. The photoperiodic gene *Ppd-D1b*, associated with the dominant vernalization gene *Vrn-D1*, influences varieties to exhibit winter or semi-winter traits, whereas spring varieties typically carry the photoperiod-insensitive alleles [11]. Cultivars with the *Ppd-D1a* allele tend to initiate flowering earlier and can mature under both long- and short-day conditions, whereas most *Ppd-D1b* cultivars do not mature under short days [12]. The influence of these genes on vernalization and photoperiodic traits may not accurately represent the winter–spring and photoperiodic characteristics of all varieties. Breeding selections and identifications for vernalization and photoperiodic traits should primarily rely on phenotypic assessments [13,14].

Rye [15], an annual or biennial herbaceous plant, boasts significant cold tolerance, drought resistance, high forage yield, and rich nutritional content, serving as a vital green fodder and grazing feed for livestock, particularly cattle and sheep, during the winter and spring seasons [15,16,17]. *Wintergrazer-70* (*Secale cereale* L. ‘*Wintergrazer-70*’) was introduced from the United States in 1978 and has since been officially registered as a variety in China. It is primarily cultivated in North China, Northeast China, parts of Northwest China, the Jianghuai River Basin, and the Yungui Plateau, making it an excellent forage crop for winter idle fields in warm temperate regions [18,19]. *Ganyin No1* (*Secale cereale* L. ‘*Ganyin No1*’) was a new variety screened from *Wintergrazer-70* by spring planting in high-altitude areas of the Qinghai–Tibet Plateau [20]. This variety is characterized by high yield, cold resistance, disease tolerance, a short growth period, and the ability to thrive in low fertility. It exhibits broad adaptability and is particularly suited for spring planting in alpine regions ranging from 2000 to 4700 m in altitude [21,22,23,24].

This study aimed to determine the spring and winter characteristics of *Ganyin No1* and establish the optimal sowing time above 3000 m a.s.l. in Tibet. Field planting trials were conducted in Linzhou County, within the Tibet Autonomous Region of China. Subsequently, the spring and winter characteristics of *Ganyin No1* were identified in Henan Province. Subsequently, genes associated with vernalization regulation and the photoperiod were screened using RNA sequencing and RT-PCR data. These findings provide a solid foundation for further research into the vernalization process and the elucidation of the molecular mechanisms underlying rye’s seasonal traits.

## 2. Materials and Methods

### 2.1. Test Site Profile

The field experiment was conducted at the Linzhou Grassland Ecosystem Observation and Research Station of the Tibet Autonomous Region (91°11′ E, 29°54′ N), situated in Lhasa City. The station is located in the Lhasa River Valley’s agricultural region, central Tibet, at an elevation of 3759 m above sea level. The region has a plateau temperate monsoon semi-arid climate, with an average annual temperature of 5.8 °C and annual rainfall ranging from 300 to 510 mm. Precipitation predominantly occurs between June and September, and the frost-free period is approximately 100–120 days.

The springiness and winterness identification site was located in the Cognition Park of Longzihu Campus, Henan Agricultural University, Zhengzhou City, Henan Province (113°82′ E, 34°79′ N). Located at an elevation of 84 m above sea level, this site experiences a continental monsoon climate typical of the northern temperate zone, characterized by frequent shifts between cold and warm air masses. The region experiences distinct seasons—spring, summer, autumn, and winter—with an average annual temperature of 15.6 °C. Precipitation averages approximately 1100 mm annually, predominantly in the summer season.

### 2.2. Experimental Materials

The study utilized *Wintergrazer-70* and *Ganyin No1* rye as experimental materials, sourced from the Lhasa Agro-Ecological Experimental Station, Chinese Academy of Sciences. The test fertilizer was a spring barley or winter wheat-specific compound fertilizer, with a total nutrient content of at least 45% (N-P_2_O_5_-K_2_O = 22-13-10), produced by China Qinghai Province Golmud city Golmud Shengnong Compound Fertilizer Co., Ltd.

### 2.3. Field Conditions

The 2021 sowing trial employed a randomized block design, following the principles of rigorous experimental design and statistical analysis. In 2021, five sowing treatments were established on different dates: 26 June (Group A), 6 July (Group B), 16 July (Group C), 26 July (Group D), and 6 August (Group E). Each treatment was replicated three times, with experimental plots measuring 16 m^2^ (4 m × 4 m). The experiment utilized line seeding with row spacings ranging from 23 to 25 cm. A basal fertilizer containing 300 kg/hm^2^ of compound fertilizer was applied. Standard irrigation and weeding practices were employed.

The springiness and winterness identification tests were conducted across eight dates in 2023: 18 February, 25 February, 4 March, 11 March, 15 March, 19 March, 23 March, and 27 March. Each treatment comprised three replicates. On 27 April, five leaf samples from each treatment group were randomly collected and sent to BGI for RNA sequencing. The experiment utilized line seeding with a row spacing of 25 cm. Diammonium phosphate was applied at a rate of 300 kg/hm^2^ as the base fertilizer. Standard irrigation and weeding practices were employed.

### 2.4. Content and Method of Determination

Forage yield (fresh): The fresh weight yield per 1 m^2^ of grass was determined by randomly sampling each plot. Samples were collected at a height of 2 cm to 3 cm above the ground during mowing.

Fresh/dry ratio: Thirty plants from each plot were randomly sampled, cut at a height of 2 cm to 3 cm above the ground, weighed for fresh weight, and then baked in a 65 °C oven for 48 h until a constant weight was achieved. The dry weight was then measured to calculate the fresh/dry ratio.

(1) $$\mathrm{Fresh}/\mathrm{dry}\;\mathrm{ratio}\;=\frac{\;30\;\mathrm{plants}\;\mathrm{fresh}\;\mathrm{weight}}{30\;\mathrm{plants}\;\mathrm{dry}\;\mathrm{weight}}$$

Hay yield: The water content of the forage was determined using the fresh/dry ratio, and the hay yield was calculated based on its moisture content.

(2) $$1\;m^{2}\;\mathrm{hay}\;\mathrm{yield}=\frac{1\;m^{2}\;\mathrm{fresh}\;\mathrm{grass}\;\mathrm{yield}\;\times 30\;\mathrm{plants}\;\mathrm{dry}\;\mathrm{weight}}{30\;\mathrm{plants}\;\mathrm{fresh}\;\mathrm{weight}}$$

Spring sowing heading rate: A primary indicator for assessing the winter–spring characteristics of wheat varieties.

(3) $$\mathrm{Heading}\;\mathrm{rate}\;\mathrm{of}\;\mathrm{spring}\;\mathrm{sowing}=\frac{\mathrm{number}\;\mathrm{of}\;\mathrm{heading}}{\mathrm{maximum}\;\mathrm{total}\;\mathrm{number}\;\mathrm{of}\;\mathrm{stems}}\times 100\%$$

RNA-seq was employed to sequence the cDNA library of rye using the BDA DNBSEQ platform. High-quality reads were obtained using the filtering software SOAPnuke v1.4.0, which removed reads with low quality, contaminated joints, and a high proportion of unknown bases (Ns). The clean reads were assembled de novo using Trinity v2.0.6, and the transcripts were clustered using TGICL to eliminate redundancy, resulting in the rye Unigene library. The quality of the assembled transcripts was evaluated using the single-copy orthologous gene database BUSCO. Candidate coding regions within the Unigene library were identified using TransDecoder v3.0.1 software, and homologous sequences from the Pfam protein database and HMMs from the Hmmscan were aligned using BLAST to predict the coding regions.

The qRT-PCR analysis employed actin as the internal reference gene. The gene-specific primers are detailed in Supplementary Table S4. The Roche 480 system (Roche, Switzerland) was employed for this study. The qRT-PCR assay was performed using the PerfectStart^TM^ M Green qPCR SuperMix kit, sourced from All-Gold Biotechnology Co., Ltd. (Beijing, China). The PCR program was as follows: initial denaturation at 94 °C for 30 s; denaturation at 94 °C for 5 s, annealing at 60 °C for 15 s, and extension at 72 °C for 15 s, for 45 cycles; and final extension at 50 °C for 10 s, followed by a temperature ramp from 60 °C to 95 °C to plot a melting curve. Relative gene expression was quantified using the 2^−ΔΔCt^ method following four replicates.

The RNA extraction and cDNA synthesis were performed using the Eastep^®^ Super total RNA kit and GoScript^TM^ Reverse Transcription kit, both provided by Promeg Bioproducts Co., Ltd. (Shanghai, China). Data analysis was conducted using Excel and R, with GraphPad Prism 8, RStudio 4.3.2, and other software employed for visualization.

## 3. Results

### 3.1. The Manipulation of Sowing Dates Significantly Influenced the Forage Yield of Rye

**Figure 1:**
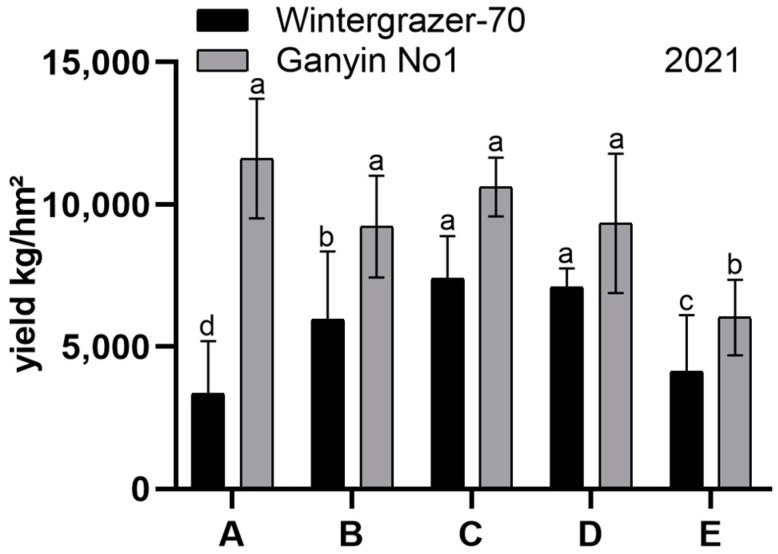
The test results of diverse planting periods; 2021 sowing date trial. Group A: 26 June, Group B: 6 July, Group C: 16 July, Group D: 26 July, Group E: 6 August. Error bars represent the standard errors of the means (SEMs) calculated from three biological replicates. The Duncan multiple range test was utilized to analyze the data and identify significant differences among treatment means. Different letters indicate a significant difference (*p* < 0.05).

Following the extended sowing period in 2021, *Wintergrazer-70*’s hay yield initially increased and subsequently decreased, whereas *Ganyin No1*’s yield exhibited a pattern of initial decline, followed by an increase, and ultimately a decrease. *Ganyin No1*’s yield was higher than *Wintergrazer-70*’s, yet the difference was not statistically significant across all groups except for Group A (*p* < 0.05, Figure 1). *Wintergrazer-70* remained in the tillering stage, while *Ganyin No1* progressed to the jointing stage. The field trials conducted in Tibet suggested a potential mutation in *Ganyin No1*, indicating its transition to a spring variety.

### 3.2. The Identification of Winter–Spring Type Indicated That Ganyin No1 Rye Is a Spring Type

**Figure 2:**
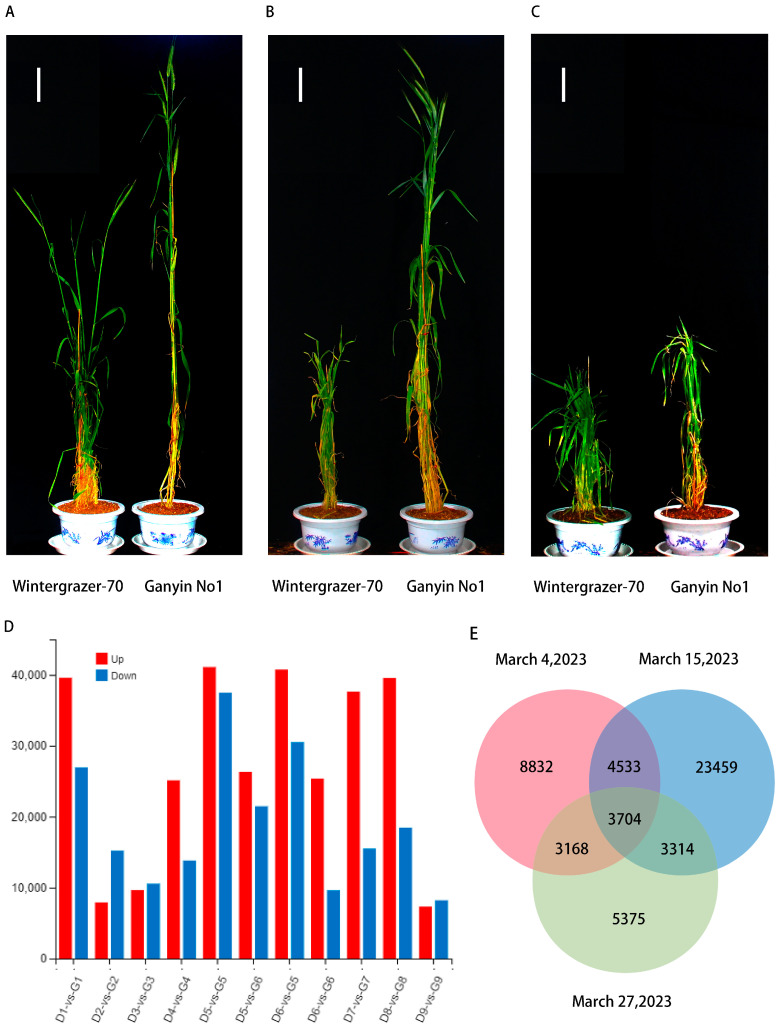
The initial investigation of field representative plant phenotype and RNA data. (**A**) Representative plant phenotype map on 4 March; (**B**) representative plant phenotype map on 15 March; (**C**) representative plant phenotype map on 27 March. *Wintergrazer-70* is located on the left-hand side, whereas *Ganyin No1* can be found on the right-hand side. (**D**) The color red indicates up-regulation, whereas blue signifies down-regulation. The *x*-axis represents the quantity of processing groups. D1-vs.-G1: 18 February processing group, D2-vs.-G2: 25 February processing group, D3-vs.-G3: 4 March processing group, D4-vs.-G4: 11 March processing group, D5-vs.-G5: 15 March processing group, D5-vs.-G6: 15 March processing group, D6-vs.-G5: 15 March processing group, D6-vs.-G6: 15 March processing group, D7-vs.-G7: 19 March processing group, D8-vs.-G8: 23 March processing group, D9-vs.-G9: 27 March processing group. (**E**) The Venn diagram depicts the distinct patterns of gene expression observed among the experimental groups treated on 4 March, 15 March, and 27 March.

In 2021, *Wintergrazer-70* remained in the tillering stage while *Ganyin No1* progressed to the jointing stage. The field trials conducted in Tibet suggested a potential mutation of *Ganyin No1* into a spring variety. Winter–spring identification tests for *Ganyin No1* and *Wintergrazer-70* were conducted in Henan to confirm whether *Ganyin No1* is a spring cultivar. Wheat cultivars that reach the jointing stage after sowing in Henan Province on 15 March (the second sowing period, with an average temperature of 7 °C on the second day) are typically classified as spring cultivars, whereas those that do not are considered winter cultivars. The results indicated that the *Wintergrazer-70* rye treatment group reached the jointing stage on 18 and 25 February and 4, 11 and 15 March but not on 19, 23, or 27 March. In contrast, all treatment groups of *Ganyin No1* rye reached the jointing stage. Utilizing the spring sowing identification protocol, *Ganyin No1* rye was classified as a spring variety (Table 1, Figure 2A–C).

### 3.3. Differential Gene Expression Analysis

Differential gene expression analysis was performed to identify genes that were differentially expressed between *Ganyin No1* and *Wintergrazer-70*. The analysis was conducted with a screening criterion of a Fold Change ≥ 2.00 and a False Discovery Rate (FDR) ≤ 0.001. Leaf sampling was performed on 27 April for subsequent RNA sequencing (refer to Supplementary Tables S1–S3). The comparison on 4 March revealed 20,236 differentially expressed genes between *Ganyin No1* and *Wintergrazer-70*.

Intersection analysis was performed on the differentially expressed genes from 4 March, 15 March, and 29 March 29. A total of 4533 genes showed differential expression common to both 4 March and 15 March. Moreover, 3168 genes exhibited differential expression between 4 March and 27 March, and 3314 genes had shared differential expression patterns between 15 March and 27 March. The expression levels of 3704 genes showed significant differences across all three treatments (Figure 2E).

### 3.4. The GO Annotation and Enrichment Analysis of Differentially Expressed Genes

**Figure 3:**
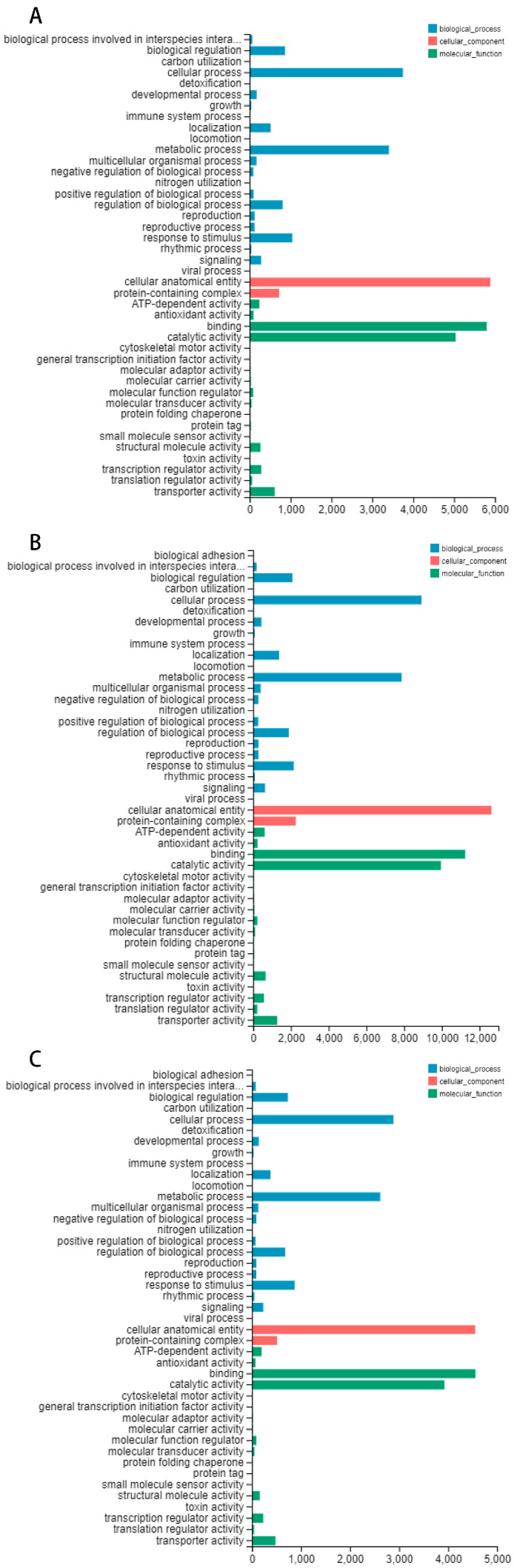
The categorization of Gene Ontology (GO) functions. (**A**) The Gene Ontology (GO) classification diagram depicting the processing group’s categorization on 4 March; (**B**) the Gene Ontology (GO) classification diagram depicting the processing group’s categorization on 15 March; (**C**) the Gene Ontology (GO) classification diagram depicting the processing group’s categorization on 27 March. Blue represents biological process, red represents the cellular component, and green represents molecular function.

In the 4 March treatment group, 10,915 differentially expressed genes were annotated in the Gene Ontology (GO) database (Figure 3A). In the 15 March treatment group, 21,564 differentially expressed genes were annotated in the Gene Ontology (GO) database (Figure 3B). In the 27 March treatment group, 8493 differentially expressed genes were annotated in the Gene Ontology (GO) database (Figure 3C). The most prevalent functional category was biological processes. The molecular function category included 18 functional groups. The cellular component category showed limited diversity, with only two functional groups identified.

**Figure 4:**
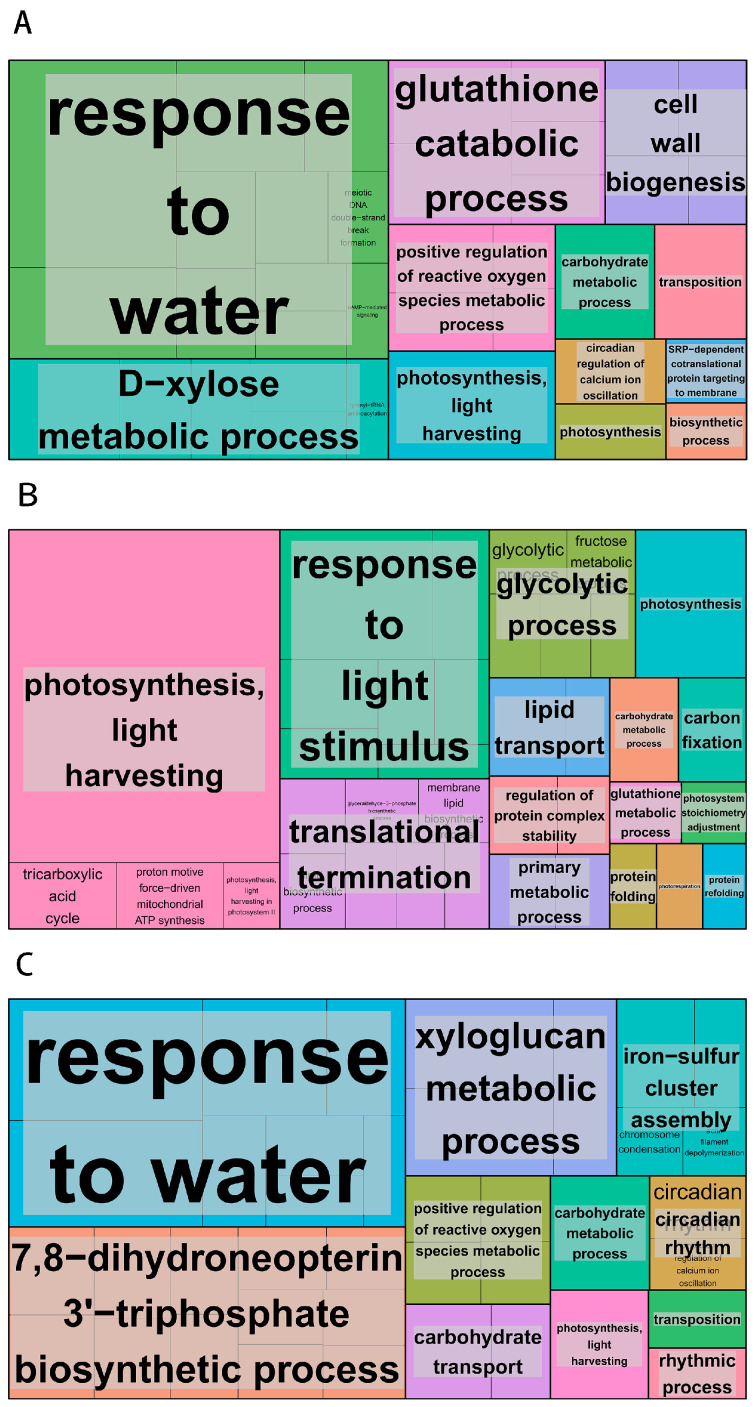
The enrichment of Gene Ontology (GO) function. (**A**) The GO enrichment map of the 4 March treatment group; (**B**) the GO enrichment map of the 15 March treatment group; (**C**) the GO enrichment map of the 27 March treatment group. The enrichment of genes becomes increasingly prominent with the expansion of the area, and distinct colors represent different pathways.

The transcriptome data underwent Gene Ontology enrichment analysis to improve the precision of target identification and enable a more accurate identification of significantly enriched functional classes [25]. On 4 March, the GO enrichment analysis indicated that the defense response category had the highest proportion (2.44%) of annotated biological processes. In the cellular components category, the integral component of the membrane represented the largest proportion (27.53%) of annotated differentially expressed genes. For molecular function classification, protein kinase activity constituted the highest proportion (5.96%) among annotated differentially expressed genes. Five significantly enriched secondary biological process classes were identified: 3005 differentially expressed genes were enriched in the integral component of the membrane; 65 in response to water; 84 in DIMBOA glucoside β-D-glucosidase activity; 99 in photosystem I; and 91 in photosynthesis and light harvesting (Figure 4A). The GO enrichment analysis performed on 15 March for the treatment group indicated that the biological process category had the highest proportion (2.38%) of annotated differentially expressed genes associated with the carbohydrate metabolic process. For cellular component classification, the chloroplast category had the highest proportion (3.33%) of annotated differentially expressed gene annotations. In the molecular function classification, the structural constituent of the ribosome represented the highest proportion (2.56%) of annotated differentially expressed genes. Five significantly enriched GO classes were identified as secondary biological process classes: photosystem II (334 differentially expressed genes), chloroplast thylakoid membrane (499 differentially expressed genes), photosystem I (297 differentially expressed genes), chlorophyll binding (286 differentially expressed genes), and photosynthesis (275 differentially expressed genes) (Figure 4B).The GO enrichment analysis performed on 27 March for the treatment group indicated that the biological process category had the highest proportion (2.77%) of annotated differentially expressed genes associated with defense response. In the cellular component classification, the integral component of the membrane represented the highest proportion (26.98%) of annotated differentially expressed genes. In the molecular function classification, ADP binding represented the most significant proportion (3.67%) of annotated differentially expressed genes. Five secondary biological process classes were identified as highly enriched within significantly enriched GO categories. These included 2291 differentially expressed genes enriched in the integral component of the membrane, 31 in glycerate dehydrogenase activity, and 48 in response to water membrane stimuli. Furthermore, hydroxypyruvate reductase activity was enriched in 31 differentially expressed genes, and the defense response was enriched in 235 differentially expressed genes (Figure 4C).

### 3.5. Differential Gene Screening Identified Genes Associated with the Vernalization Process

The GO enrichment analysis identified 26 genes associated with vernalization. These genes predominantly encode Vernalization Insensitive 3-like proteins, characterized by Oberon-like, PHD finger domain (PHD), and Fibronectin Type III (FN3) domains (refer to Supplementary Figure S1). These Vernalization Insensitive 3-like proteins feature a plant homeodomain (PHD) that suppresses the FLOWERING LOCUS C (FLC) gene family, thus inhibiting flowering during vernalization [26,27,28].

**Figure 5:**
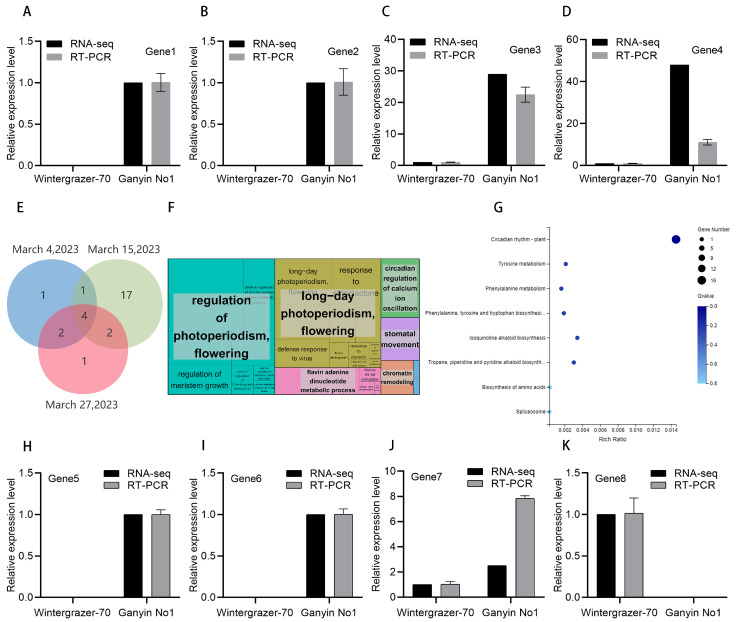
Analysis of RNA-seq results. The expression of genes was assessed using RNA-seq and RT-PCR methodologies. (**A**) Gene1: Sc22136g1_i2G5; (**B**) Gene2: Sc18554g1_i1G5; (**C**) Gene3: Sc5712g1_i3G8; (**D**) Gene4: Sc10255g1_i2G7; (**H**) Gene5: Sc128g3_i1D2; (**I**) Gene6: Sc2503g1_i7G8; (**J**) Gene7: Sc6095g1_i5G6; (**K**) Gene8: Sc7296g5_i1G3y. (**E**) The Venn diagrams depict the intersection of differentially expressed genes photoperiodic on 4 March, 15 March, and 27 March. (**F**) The GO enrichment map of differentially expressed genes under photoperiodic conditions was constructed. The enrichment of genes becomes increasingly prominent with the expansion of the area, and distinct colors represent different pathways. (**G**) KEGG-enriched bubble map illustrating differentially expressed genes in response to photoperiodic regulation. The gene RT-PCR primer sequence is shown in Supplementary Table S4. The RT-PCR results for other photoperiodic genes are presented in Supplementary Figure S2.

Subsequent screening on 15 March identified four genes with differential expression. RT-PCR analysis further confirmed significant differences in the expression levels of these genes. Specifically, *Ganyin No1* expressed Sc22136g1_i2G5 (Gene1) and Sc18554g1_i1G5 (Gene2), which were not detected in *Wintergrazer-70* (Figure 5A,B). Additionally, *Ganyin No1* showed significantly higher expression of Sc5712g1_i3G8 (Gene3) and Sc10255g1_i2G7 (Gene4) compared to *Wintergrazer-70* (Figure 5C,D). These findings suggest a possible association between these four genes and the promotion of flowering in rye, which may account for the observed differences in vernalization between the two varieties.

### 3.6. Differential Gene Screening to Genes Associated with the Photoperiodic

The GO enrichment analysis identified 144 photoperiodic genes, with 28 showing differential expression. On 4 March, treatment revealed significant differential expression in eight genes. Similarly, 24 genes exhibited differential expression in the group treated on 15 March. Additionally, nine genes displayed differential expression in the group treated on 27 March. Notably, four genes were common to all treatment groups. The GO enrichment analysis indicated significant enrichment of these differentially expressed genes in several pathways, including the regulation of photoperiod, flowering, positive regulation of long-day photoperiod, circadian regulation of calcium ion oscillation, flavin adenine dinucleotide metabolism, and response to strigolactone. The KEGG enrichment analysis revealed substantial enrichment of differentially expressed genes in multiple metabolic pathways, including the circadian rhythm plant; tyrosine metabolism; phenylalanine metabolism; biosynthesis of phenylalanine, tyrosine, and tryptophan; isoquinoline alkaloid biosynthesis; tropane, piperidine and pyridine alkaloid biosynthesis; and amino acid biosynthesis; as well as the spliceosome pathway.

Most differentially expressed genes were analyzed on 15 March. Combining RT-PCR and RNA-seq results revealed that 15 differentially expressed genes were specific to *Ganyin No1* on 15 March and were not detected in *Wintergrazer-70* (Figure 5H,I and Figure S2). The expression of one additional gene in Ganyin No1 was significantly higher than in *Wintergrazer-70* (Figure 5J). These 15 genes, along with the additional one mentioned, may be involved in promoting rye flowering. The Sc7296g5_i1G3 gene was found to be expressed in *Wintergrazer-70*, but not in *Ganyin No1* (Figure 5K). This gene may play a role in inhibiting rye flowering. It is hypothesized that these photoperiod-related genes may also contribute to the differences in vernalization between the two varieties.

### 3.7. The KEGG Annotation and Enrichment Analysis of Differentially Expressed Genes

**Figure 6:**
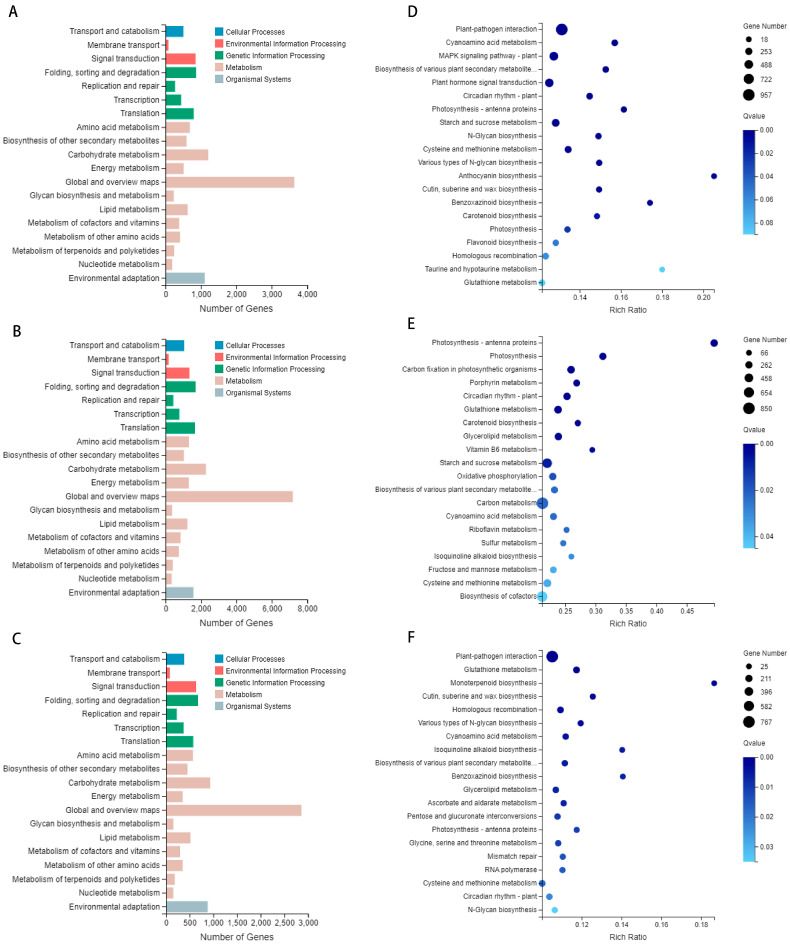
Analysis utilizing the KEGG database. (**A**) The KEGG classification diagram of the treatment group on 4 March 4; (**B**) the KEGG classification diagram of the treatment group on 15 March; (**C**) the KEGG classification diagram of the treatment group on 27 March. Blue represents cellular processes, red represents environmental information processing, green represents genetic information processing, brown represents metabolism, and gray represents organismal systems. (**D**) The KEGG enrichment bubble diagram of the treatment group on 4 March; (**E**) the KEGG enrichment bubble diagram of the treatment group on 15 March; (**F**) the KEGG enrichment bubble diagram of the treatment group on 27 March.

The KEGG database is a comprehensive resource for systematically analyzing cellular metabolic pathways and functionally characterizing gene products [29]. Annotation was performed on 4 March, 15 March, and 27 March, resulting in the annotation of 7376, 13,984, and 5741 differentially expressed genes, respectively. The majority of the annotated differentially expressed genes on these dates were predominantly associated with five specific metabolic pathways (Figure 6A–C). The cellular processes pathway was associated with a total of 507, 1055, and 386 differentially expressed genes across the three experimental conditions.

### 3.8. KEGG Enrichment Analysis

KEGG enrichment analysis of the March 4 treatment group identified a significant proportion (12.97%) of the 957 differentially expressed genes as being associated with plant–pathogen interaction, a metabolic pathway with an increased presence of these genes. Additionally, many differentially expressed genes were involved in key pathways, including the MAPK signaling pathway-plant (6.67% of 492 genes), plant hormone signal transduction (6.03% of 445 genes), and starch and sucrose metabolism (4.53% of 334 genes). Significantly, the five most enriched pathways identified were plant–pathogen interaction, cyanoamino acid metabolism, MAPK signaling pathway-plant, biosynthesis of various plant secondary metabolites, and plant hormone signal transduction (Figure 6D). The KEGG enrichment analysis for the 15 March treatment group indicated that carbon metabolism was the most enriched pathway, with 850 differentially expressed genes (6.08% of the total). Starch and sucrose metabolism followed, involving 574 differentially expressed genes (4.10%). The metabolism of cysteine and methionine was the third most enriched, with 355 differentially expressed genes (2.54%). The five significantly enriched metabolic pathways were plant–pathogen interaction, photosynthesis, carbon fixation in photosynthetic organisms, porphyrin metabolism, and circadian rhythm-plant (Figure 6E). The KEGG enrichment analysis for the 27 March comparison group identified 767 differentially expressed genes associated with plant–pathogen interaction, which constituted 13.36% of the total gene set. Additionally, glutathione metabolism was associated with differential expression in 163 genes (2.84%), and cysteine and methionine metabolism in 161 genes (2.80%). Homologous recombination also showed differential expression in 147 genes (2.56%). Significantly, the top five enriched metabolic pathways were plant–pathogen interaction; glutathione metabolism; monoterpenoid biosynthesis; cutin, suberine and wax biosynthesis; and homologous recombination (Figure 6F). The KEGG annotation and enrichment analysis identified plant–pathogen interaction as the predominant enriched pathway on 4 March and 27 March, and carbon metabolism as the main enriched pathway on 15 March. These findings highlight the substantial differences in plant–pathogen interactions during the tillering and jointing stages, with various plant hormones playing pivotal regulatory roles. A variety of distinct plant hormones facilitate diverse signaling pathways in the interactions between plants and pathogenic microorganisms, thus shaping the plant’s defense responses to various pathogens.

## 4. Discussion

The Henan wheat district test findings suggest that the spring sowing method, which uses the heading rate of stage 2 as a fundamental indicator, is reliable for assessing wheat’s winter and spring characteristics [30,31,32]. A comprehensive sequence classification of wheat spring sowing enabled us to identify the winter–spring traits in both *Wintergrazer-70* and *Ganyin No1* rye varieties. The effect of mRNA on leaf transcriptomics in *Wintergrazer-70* and *Ganyin No1* rye was examined using full-length transcriptomics over multiple planting periods. This analysis uncovered altered expression profiles for the two rye cultivars under various planting conditions. The findings indicated a higher number of differentially expressed genes in Ganyin No1 during the jointing stage and in *Wintergrazer-70* during the tillering stage. For both varieties, the number of differentially expressed genes was relatively low during both the jointing and tillering stages. The observed differences primarily stem from significant variations in physiological and biochemical processes during the same growth period, aligning with results from other studies.

The Gene Ontology (GO) annotation and enrichment analysis of differentially expressed genes revealed significant enrichments in various biological processes, including cellular and metabolic processes. A notable disparity in the growth period was observed between the identical species planted simultaneously and the mutant varieties. Mutation sites in *Ganyin No1* rye were not limited solely to the vernalization gene. The differential gene expression profiles, obtained from the GO analysis, identified genes involved in vernalization and photoperiodic regulation. This study encountered limitations, as a subset of differentially expressed genes associated with vernalization and photoperiod was not identified. Further studies using alternative databases may be warranted for screening.

Six major pathways have been identified for regulating flowering time in plants, comprising three external pathways (photoperiodic regulation, vernalization, and ambient temperature) and three endogenous pathways (autonomous, age, and gibberellin pathways) [33,34,35,36]. This study primarily examines the regulatory pathways of photoperiod and vernalization within the external signaling cascades. The vernalization treatment identified four differentially expressed genes, whereas the photoperiod treatment revealed twenty-eight. Notably, the 15 March treatment group showed a majority of these differentially expressed genes, marking it as a distinct group. Among these, genes associated with vernalization were found to promote rye flowering, and it was found that most photoperiod-related genes also contribute to flowering. However, the Sc7296g5_i1G3 gene seems to inhibit flowering. Variations in these genes are hypothesized to explain the differences in vernalization between the two varieties. The functions of these genes can be further validated, and identifying the differential sites may help address the variation between the two varieties.

The KEGG annotation and enrichment analysis identified plant–pathogen interaction as the predominantly enriched pathway among the differentially expressed genes. Plant resistance against biotrophic pathogens is primarily mediated by salicylic acid, whereas necrotrophic pathogen infections are mainly regulated by jasmonic acid and ethylene, which often exhibit antagonistic effects during plant–pathogen interactions. Nonetheless, notable differences in carbon metabolism were detected between the two rye varieties during the tillering and jointing stages, possibly attributable to variations in nutrient conversion mechanisms influencing biomass production. The growth of plants is regulated by many PGPRs through the production of auxins, gibberellins, and cytokinins. Furthermore, the observed differences in rye at the tillering or jointing stage may stem from an increased demand for auxins and other hormones.

## 5. Conclusions

In conclusion, our study on the introduction of *Wintergrazer-70* and *Ganyin No1* rye in Tibet revealed significant differences between the two varieties in terms of summer sowing. Subsequently, identification tests confirmed that *Ganyin No1* is a spring variety. RNA-seq identified 26 genes associated with the vernalization process, of which 4 were differentially expressed, all implicated in promoting vernalization. RNA-seq also identified 144 genes associated with the photoperiod, with 28 differentially expressed genes, predominantly involved in promoting flowering. However, the Sc7296g5_i1G3 (Gene8) gene was found to be associated with inhibiting flowering. Its remarkable adaptability to environmental conditions, along with beneficial traits such as resistance to barrenness, lodging, and rapid growth, make *Ganyin No1* rye highly suitable for cultivation in the challenging Qinghai–Tibet Plateau region.

## Figures and Tables

**Table 1:** Identification during the winter–spring period.

Planting Season	Species Name	Growth Pattern	Seedling-Heading Period/d	Heading Rate/%
18 February	*Wintergrazer-70*	3	69	1.3
	*Ganyin No1*	3	42	63.4
25 February	*Wintergrazer-70*	3	67	0.9
	*Ganyin No1*	3	42	61.8
4 March	*Wintergrazer-70*	3	68	0.8
	*Ganyin No1*	3	41	62.7
11 March	*Wintergrazer-70*	3	68	1.1
	*Ganyin No1*	3	42	63.2
5 March	*Wintergrazer-70*	3	67	1
	*Ganyin No1*	3	41	63.8
19 March	*Wintergrazer-70*	3		
	*Ganyin No1*	3	40	63.1
23 March	*Wintergrazer-70*	3		
	*Ganyin No1*	3	40	61.3
27 March	*Wintergrazer-70*	3		
	*Ganyin No1*	3	40	62.9

## Data Availability

The data presented in this study are available upon request from the corresponding author. The data are not publicly available due to privacy restrictions.

## Supplementary Materials

The following supporting information can be downloaded at: https://www.mdpi.com/article/10.3390/genes15050572/s1, Figure S1: The prediction of protein domains in genes associated with the vernalization process; Figure S2: The expression of genes related to photoperiod were assessed using RNA-seq and RT-PCR methodologies; Table S1: Prediction results and data assembly; Table S2: Unigene; Table S3: Functional database annotation; Table S4: RT-PCR primer sequence.

## References

[1] Liu X. (2020). Evaluation Ofmain Agronomic Traits and Identification of Springness and Winterness in *Secale cereale* subsp. Segetale. Master’s Thesis.

[2] Sun J., Zhang K., Yang Z., Qiu J., Zhang X. (2023). Study on Distinguishing Wheat Growth Habits by Spring Sowing in Different Periods. Mod. Agric. Sci. Technol..

[3] Chen L. (2017). The Relationship between Winter-Spring Habit and Growth Period in Wheat Varieties in the South of Huanghuai Region. Master’s Thesis.

[4] Niu D., Gao Z., Cui B., Zhang Y., He Y. (2024). A Molecular Mechanism for Embryonic Resetting of Winter Memory and Restoration of Winter Annual Growth Habit in Wheat. Nat. Plants.

[5] Trevaskis B. (2010). The Central Role of the Vernalization1 Gene in the Vernalization Response of Cereals. Funct. Plant Biol..

[6] Yan L., Helguera M., Kato K., Fukuyama S., Sherman J., Dubcovsky J. (2004). Allelic variation at the VRN-1 promoter region in polyploid wheat. Theor. Appl. Genet..

[7] Amo A., Serikbay D., Song L., Chen L., Hu Y.-G. (2022). Vernalization and Photoperiod Alleles Greatly Affected Phenological and Agronomic Traits in Bread Wheat under Autumn and Spring Sowing Conditions. Crop Environ..

[8] Song K., Shi T., Jia R., Wang X., Huang X., Xiao Q. (2023). Breeding of a High-Yield, Multi Resistance and Semi-Winter Wheat Variety Nongmai 168. China Seed Ind..

[9] Jiang Y., Huang L.-Z., Hu Y.-G. (2010). Distribution of Vernalization Genes in Chinese Wheat Landraces and Their Relationship with Winter Hardness. Sci. Agric. Sin..

[10] Zhao J., Liu H., Sun L., Zhao Y., Yang K., Hu M., Li H., Fu Y., Gu Y., Zhang Y. (2022). Composition and Distribution of Wheat Vrn Genes and Their Relationship with Vernalization in the Northern China Winter Wheat Region. Acta Agric. Boreali-Sin..

[11] Cao W., Liu S., Yang Q., Zhang W. (2016). Characteristics of Vernalization Gene and Photoperiod Gene and Their Relationship with Winter Hardness Revealed by Sts Markers. Mol. Plant Breed..

[12] Yang F.-P., Xia X.-C., Zhang Y., Zhang X.-K., Liu J.-J., Tang J.-W., Yang X.-M., Zhang J.-R., Liu Q., Li S.-Z. (2012). Distribution of Allelic Variation for Vernalization, Photoperiod, and Dwarfing Genes and Their Effects on Growth Period and Plant Height among Cultivars from Major Wheat Producing Countries. Acta Agron. Sin..

[13] Zheng S. (2019). Genetic Variation Ofvernalization Characteristic and Photoperiod Characteristics Ofwheat Varieties in Henan Province. Master’s Thesis.

[14] Zhang S., Xu W., Fang Y., Li Z., LI C., Zhang Y., Qi X. (2019). Vernalization Characteristic and Photoperiod Characteristics of Wheat Varieties in Henan Province. J. Triticeae Crops.

[15] Huddell A.M., Thapa R., Marcillo G.S., Abendroth L.J., Ackroyd V.J., Armstrong S.D., Asmita G., Bagavathiannan M.V., Balkcom K.S., Basche A. (2024). U.S. Cereal Rye Winter Cover Crop Growth Database. Sci. Data.

[16] Zhao S., Han X., Zhu Y., Han Y., Liu H., Chen Z., Li H., Wang D., Tian C., Yuan Y. (2024). Crispr/Casφ2-Mediated Gene Editing in Wheat and Rye. J. Integr. Plant Biol..

[17] Guo J., TIan X., Du W. (2017). Study on Yield of Rye Seed and Its Constituent Factors. Gansu Agric. Sci. Technol..

[18] Chen L. (2004). Wintergrazer-70 Rye Cultivation and Utilization Technology. China Cattle Sci..

[19] Huang H. (2000). Cultivation Techniques and Utilization of Wintergrazer-70 Rye. Mod. Anim. Husb..

[20] Meng X., Han T., Han Y. (2015). Breeding and Variety Characteristics of Ganyin No1 Rye. Gansu Anim. Husb. Vet. Med..

[21] Fu X., Pan Z., Meng X., Cao Y., Li C., Zhang Q. (2017). The Relationship of Agronomic Traits and Fresh Forage Yield of *Secale cereale* L. ‘Ganyin No1’. Acta Agrestia Sin..

[22] Meng X., Han T., Yu L., Fu X. (2016). Effects Ofseeding Rate, Row Spacing and Fertilization on the Aboveground Biomass of *Secale cereale* L. ‘Ganyin No1’. China Herbiv. Sci..

[23] Meng X., Yu L., Zhang S., Yang H. (2017). Adaptability Evaluation of Ganyin No1 Rye in Alpine Region. Grassl. Turf.

[24] Yu L., Meng X., Li X., Chen X. (2015). Evaluation on the Production Performance Ofsecale Cereale L. ‘Ganyin No 1’ and It’s Supplementary Feeding Value for Grazing Sheep in Alpine Region. China Herbiv. Sci..

[25] Chen L., Zhu Y., Lu A., Wu Y., Ji H., Chen Z., Wu S., Zhai J. (2023). The Analysis of Expression Patterns of Heat Stress-Related Genes in Two Species of Calanthe R. Br. J. Plant Genet. Resour..

[26] Kim D.H., Sung S. (2013). Coordination of the Vernalization Response through a Vin3 and Flc Gene Family Regulatory Network in Arabidopsis. Plant Cell.

[27] Greb T., Mylne J.S., Crevillen P., Geraldo N., An H., Gendall A.R., Dean C. (2007). The Phd Finger Protein Vrn5 Functions in the Epigenetic Silencing of Arabidopsis Flc. Curr. Biol..

[28] Gaurav N., Kutateladze T.G. (2023). Non-Histone Binding Functions of Phd Fingers. Trends Biochem. Sci..

[29] Kanehisa M., Sato Y., Kawashima M. (2022). Kegg Mapping Tools for Uncovering Hidden Features in Biological Data. Protein Sci..

[30] Meng L.-M., Yang Z.-G., Zhang K., Sun J.-W., Ji T.-H. (2020). Application of Winterness and Springness Identification of Wheat on Regional Trial. Hubei Agric. Sci..

[31] Wang R., Yang X., Li X., Ou X. (2013). Identification of Character of Springness and Winterness of Wheat by Optimum Sowing Date in North of Henan Province. J. Henan Inst. Sci. Technol..

[32] Yang Z.-G., Sun J.-W., Zhang K., Meng L.-M. (2022). Application and Practice of Identification of Winter-Spring Character of Wheat in Regional Test. Hubei Agric. Sci..

[33] Ding Y., Wang M.Y., Yang D.H., Hao D.C., Li W.S., Ling P., Xie S.Q. (2023). Transcriptome Analysis of Flower Colour Reveals the Correlation between Snp and Differential Expression Genes in Phalaenopsis. Genes Genom..

[34] Izawa T. (2021). What Is Going on with the Hormonal Control of Flowering in Plants?. Plant J..

[35] Fornara F., de Montaigu A., Coupland G. (2010). Snapshot: Control of Flowering in Arabidopsis. Cell.

[36] Kinoshita A., Richter R. (2020). Genetic and Molecular Basis of Floral Induction in Arabidopsis Thaliana. J. Exp. Bot..

