# Supplementary material for: Identification of a Rye Spring Mutant Derived from a Winter Rye Variety by High-Altitude Environment Screening Using RNA Sequencing Technology
Source: Genes (Basel). 2024 Apr 29;15(5):572. doi: 10.3390/genes15050572 (PMC11121467; doi:10.3390/genes15050572)
Supplement: Supplementary file 1 [file genes-15-00572-s001.zip › genes-2946299-supplementary.pdf]

Supplementary Materials

Table S1. Prediction results and data assembly

Disposal	Total Raw Reads (M)	Total Clean Reads (M)	Clean Reads Q20 (%)	Clean Reads Q30 (%)	Clean Reads Ratio (%)
D1	43.69	42.52	97.84	92.69	97.31
D2	43.69	42.45	97.96	93.11	97.17
D3	43.69	42.4	97.9	92.91	97.05
D4	43.69	42.46	98.01	93.27	97.18
D5	43.69	42.12	98.03	93.35	96.41
D6	43.69	42.43	98.02	93.28	97.1
D7	43.69	42.25	97.37	91.8	96.71
D8	43.69	42.27	98.08	93.5	96.76
D9	43.69	42.14	97.57	91.9	96.44
G1	43.69	42.18	98.03	93.35	96.55
G2	43.69	42.23	98	93.28	96.65
G3	43.69	42.23	97.88	92.84	96.65
G4	43.69	42.22	97.85	92.72	96.64
G5	43.69	42.32	97.97	93.09	96.86
G6	43.69	42.64	97.95	93.11	97.59
G7	43.69	42.1	98.07	93.49	96.36
G8	43.69	42.3	98.03	93.34	96.83
G9	43.69	42.37	97.94	93.07	96.99

The rye leaf transcriptome was sequenced using the BGI interactive reporting system, resulting in the generation of a comprehensive dataset of full-length transcripts. Initially, a read count of 43.69M was obtained, which was subsequently refined by filtering out reads containing adapters, those with unknown base N content exceeding 5%, and low-quality reads. As a result, the clean read count reached 42.1M or higher. Notably, the sequencing data exhibited exceptional accuracy with Q20 values exceeding 97% and Q30 values surpassing 91%, thereby satisfying the prerequisites for constructing a robust database.

Table S2. Unigene

Processing	Total Number	Total Length	Mean Length	N50	N70	N90	GC (%)
D1	47187	45439129	962	1679	1093	407	49.04
D2	51274	48038108	936	1631	1051	394	49.77
D3	47168	45472614	964	1663	1090	409	50.05
D4	47459	45089974	950	1645	1071	398	50.13
D5	70575	55209047	782	1334	813	314	50.57
D6	44815	42341601	944	1637	1067	398	50.26
D7	41437	39936026	963	1632	1079	418	50.37
D8	41520	39308312	946	1617	1060	404	50.75
D9	43748	41426074	946	1627	1071	403	50.32
G1	80410	59490684	739	1249	751	297	50.71
G2	43227	41354632	956	1646	1081	405	50.52
G3	45121	44481790	985	1677	1111	434	50.27
G4	58286	46170899	792	1417	835	306	50.87
G5	77022	56156550	729	1229	731	293	51.07
G6	57282	46906254	818	1460	885	319	50.41
G7	75041	56249867	749	1276	757	301	51.07
G8	77583	57690832	743	1268	747	296	51.31
G9	42706	41502197	971	1665	1096	422	50.54

The clean reads (PCR duplicates removed to enhance assembly efficiency) were de novo assembled using Trinity, followed by transcript clustering with CD-HIT to eliminate redundancy and obtain Unigene.

Table S3. Functional database annotation

Values	Total	NR	NT	Swiss-prot	KEGG	KOG	Pfam	GO	Interse-ction	Overall
Number	274,923	195,773	211,282	120,950	127,966	100,138	115,445	128,492	46,612	229,790
Percentage	100%	71.21%	76.85%	43.99%	46.55%	36.42%	41.99%	46.74%	16.95%	83.58%

Table S4. RT-PCR primer sequence

Gene	Forward primer (5' – 3')	Reverse primer (5' – 3')
Actin	CGTGTTGGATTCTGGTGATG	AGCCACATATGCGAGCTTCT
Gene 1 (Sc22136g1_i2G5)	ATTACATCGTCTTCCCTTGTTATCATC	TGCTCTTCCAGTACCATAGCTTATAAC
Gene 2 (Sc18554g1_i1G5)	GTCGAGCGGAGGAGATCTGTC	TCATCAGCATGTCCACTTTCTTCAG
Gene 3 (Sc5712g1_i3G8)	ATTATTGAACCTGCTAAATGCCGATTG	GCTTTGACAATGCACGCACAAG
Gene 4 (Sc10255g1_i2G7)	ACCTCGATTCTCTGCCTCTATGC	TCCACCAGTCTCCTTACCATTCTC
Gene 5 (Sc128g3_i1D2)	GCCGTACTACTATCCGCCGATG	CCGTAAACCCTTGCCCTTCCTC
Gene 6 (Sc2503g1_i7G8)	AACGCTTATCAGGCAATGTCACAAG	AGTCGCTTCTTCCTGCTGAGTAAC
Gene 7 (Sc6095g1_i5G6)	GCTCACAACAATGTCTCCAGAACC	TCTTGCCACCTTTCCGATCCTTG
Gene 8 (Sc7296g5_i1G3)	GGTTAGCAGCGGAGGGATATGTC	TGACTGGTTAGCAGGCATGTGG
Gene 9 (Sc1035g1_i1G3)	GGAGATCAACGGCGGCTACC	AGGACGCTGCTGCTTCTTCTG
Gene 10 (Sc1067g1_i5D8)	GCCTACGTCGCCTACCTTTTC	TCGTCTCCGCCCTCGATCTC
Gene 11 (Sc11059g1_i5G7)	AGTTGCTAGACGATCACCTGAAGAC	GAAGAACAAACGAGCCATCCCAAG
Gene 12 (Sc1438g1_i1G9)	ACCTCCGCCTCGATCTCAATG	CAGCCGCTTGCCGAATGC
Gene 13 (Sc155g1_i1G5)	GCTGATGAGGATGAGTACCGATGC	TGACTTTGCCGTATTTCGAGAATGC
Gene 14 (Sc15708g1_i1G6)	CTCGCCTTATACCTGAGCATATTCG	GCCAGAAGCCAGTAGAAGTAATCG
Gene 15 (Sc10106g1_i4D7)	GGGTCTTGGTCGGAAAGCTAAC	GGTATATGGCGATTCGTGGTTGAG
Gene 16 (Sc1528g1_i2D7)	CGCCTCCAAGCTGCTGTCC	AGGAGAGGATGAGCGCGAAGG
Gene 17 (Sc1630g1_i1G6)	CCCAAGATGCTCGCCGTGATG	TGCCAGCACCGCCACCTC
Gene 18 (Sc10133g2_i1G3)	TCCAAGTCCAAGCGATCCCAAC	TCCAAGTCCAAGCGATCCCAAC
Gene 19 (Sc19522g1_i3G1)	TCCAAGTCCAAGCGATCCCAAC	CTGTCCGAACGAGGCACCAG

Gene 20 (Sc8492g1_i5D3)	AGCATGTCGGTGTTCCTGTCTG	CGAACGCCCTTCTTGGTTTGTC
Gene 21 (Sc9111g1_i2D3)	AAGACTCGGGCTCCATATCAAACTC	TCCTGACGACCCAACTGAATAACC
Gene 22 (Sc10133g3_i1G3)	AGTATCTTTCGGGACTGAGGTTGTG	GCTGCTGGAAGAGGAGGAACAC
Gene 23 (Sc128g3_i1D2)	AAGACTCGGGCTCCATATCAAACTC	TCCTGACGACCCAACTGAATAACC
Gene 24 (Sc519g1_i1D6)	GTGCCGTCTATCGCCTACCATC	GTGCCGTCTATCGCCTACCATC
Gene 25 (Sc3962g6_i1D4)	TGGTTATTCAGTTGGGTCGTCAGG	GGCGGCAGGTCTCTTGGATG
Gene 26 (Sc705g1_i3D7)	TCCTCCGTGACATCTTGAACAGTG	CGTTGCTCGCCTTGAATGATCTTAG
Gene 27 (Sc10532g1_i1G5)	CCTTCGTCCGTACCACCAATCTC	TCTCATTTCCGCCGACCTCAAC
Gene 28 (Sc188g1_i1G8)	ATGGGAAATGGATCGATGGTCTCC	CAAAGTTTGTGCCTGTTTCTGCTG
Gene 29 (Sc188g1_i3G8)	GTCAAATGGGAAGTGGATTGATGGG	CAAAGTTTGTGCCTGTTTCTGCTG
Gene 30 (Sc2014g1_i6G7)	GCCGTACTACTATCCGCCGATG	CCGTAAACCCTTGCCCTTCCTC
Gene 31 (Sc20227g1_i1G5)	GCTCTTGGCTGGGTGGATCG	CCTCCTGGTGCTGTGCTTGG
Gene 32 (Sc2063g1_i1G1)	CCGAACGCTTATCAGGCAATGTC	TCGCTTCTTCCTGCTGAGTAACC
Gene 33 (Sc2153g1_i2G1)	TAGGGTTTCTTCACACAGGGACAAG	GACTCAGCAGCATTCTCATTCCTTC
Gene 34 (Sc2979g1_i2G4)	CTTGGCTGTTTAAGAACCTCGGAAC	GATTTCTGGTCACTTTGTGGGATGC
Gene 35 (Sc4269g1_i7G5)	ACAACCTCCCTCCGGCCAAG	GCCGTACCGCTCCTTGAACC
Gene 36 (Sc4626g1_i1G8)	GGAAATGAACCTGAACGGAAGCC	ATGCCCAAAGGTGCGGTTAGC
Gene 37 (Sc7428g1_i4G5)	CGTGGATGGCATGAATCAAGGAAG	TCGTAGCTGCTGGATGGTTGTG
Gene 38 (Sc91g1_i7G4)	ATGGGAAATGGATCGATGGTCTCC	CAAAGTTTGTGCCTGTTTCTGCTG

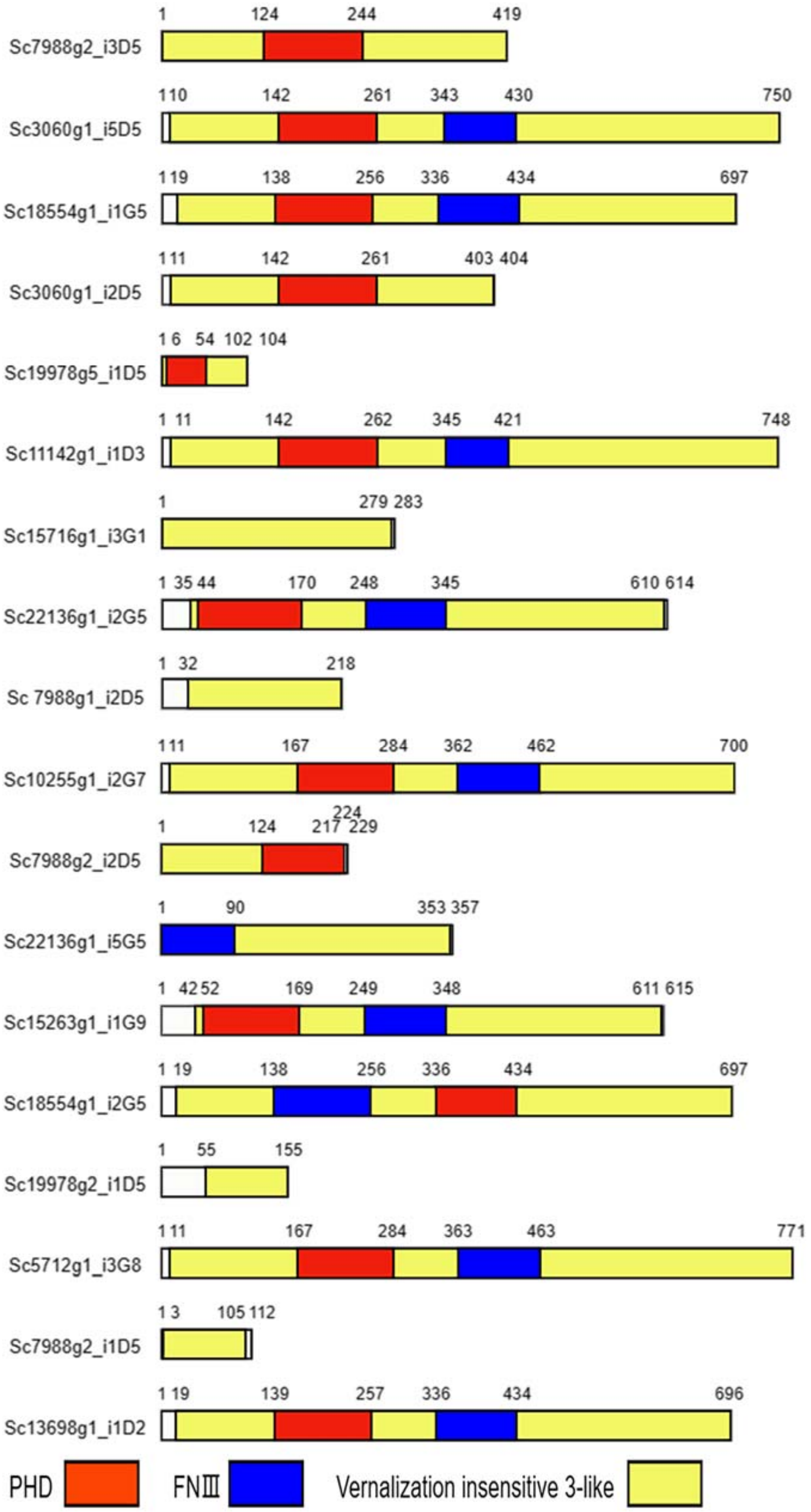

Figure S1. The prediction of protein domains in genes associated with the vernalization process. Red represents PHD、Blue represents FN||| 、Yellow represents Vernalization insensitive 3-like.

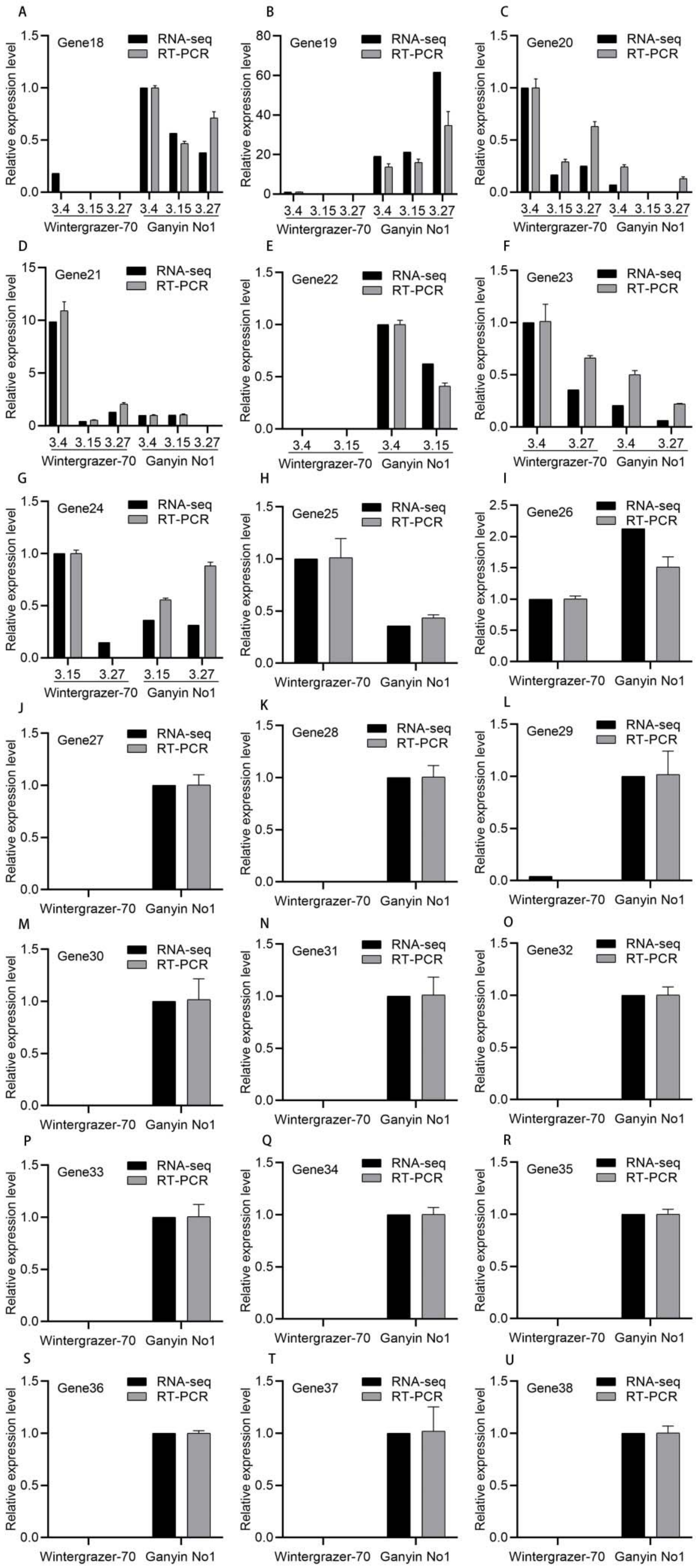
A
Gene18
RNA-seq
RT-PCR
Relative expression level
3.4 3.15 3.27 3.4 3.15 3.27
Wintergrazer-70 Ganyin No1
B
Gene19
C
Gene20
D
Gene21
E
Gene22
F
Gene23
G
Gene24
H
Gene25
I
Gene26
J
Gene27
K
Gene28
L
Gene29
M
Gene30
N
Gene31
O
Gene32
P
Gene33
Q
Gene34
R
Gene35
S
Gene36
T
Gene37
U
Gene38

Figure S2. The expression of genes related to photoperiod were assessed using RNA-seq and RT-PCR methodologies

(A)Gene18: Sc10133g2_i1G3; (B)Gene19: Sc19522g1_i3G1; (C)Gene20: Sc8492g1_i5D3; (D)Gene21: Sc9111g1_i2D3; (E)Gene22: Sc10133g3_i1G3; (F)Gene23: Sc128g3_i1D2; (G)Gene24: Sc519g1_i1D6; (H)Gene25: Sc3962g6_i1D4; (I)Gene26: Sc705g1_i3D7; (J)Gene27: Sc10532g1_i1G5; (K)Gene28: Sc188g1_i1G8; (L)Gene29: Sc188g1_i3G8; (M)Gene30: Sc2014g1_i6G7; (N)Gene31: Sc20227g1_i1G5; (O)Gene32: Sc2063g1_i1G1; (P)Gene33: Sc2153g1_i2G1; (Q)Gene34: Sc2979g1_i2G4; (R)Gene35: Sc4269g1_i7G5; (S)Gene36: Sc4626g1_i1G8; (T)Gene37: Sc7428g1_i4G5; (U)Gene38: Sc91g1_i7G4 . The primer sequences for gene RT-PCR can be found in the supplementary Table 5.
